# Recent Progress in Solution Structure Studies of Photosynthetic Proteins Using Small-Angle Scattering Methods

**DOI:** 10.3390/molecules28217414

**Published:** 2023-11-03

**Authors:** Maksym Golub, Jörg Pieper

**Affiliations:** Institute of Physics, University of Tartu, Wilhelm Ostwald Str. 1, 50411 Tartu, Estonia; maksym.golub@ut.ee

**Keywords:** small-angle neutron scattering, solution structure, contrast variation, deuteration, detergent belt

## Abstract

Utilized for gaining structural insights, small-angle neutron and X-ray scattering techniques (SANS and SAXS, respectively) enable an examination of biomolecules, including photosynthetic pigment-protein complexes, in solution at physiological temperatures. These methods can be seen as instrumental bridges between the high-resolution structural information achieved by crystallography or cryo-electron microscopy and functional explorations conducted in a solution state. The review starts with a comprehensive overview about the fundamental principles and applications of SANS and SAXS, with a particular focus on the recent advancements permitting to enhance the efficiency of these techniques in photosynthesis research. Among the recent developments discussed are: (i) the advent of novel modeling tools whereby a direct connection between SANS and SAXS data and high-resolution structures is created; (ii) the employment of selective deuteration, which is utilized to enhance spatial selectivity and contrast matching; (iii) the potential symbioses with molecular dynamics simulations; and (iv) the amalgamations with functional studies that are conducted to unearth structure-function relationships. Finally, reference is made to time-resolved SANS/SAXS experiments, which enable the monitoring of large-scale structural transformations of proteins in a real-time framework.

## 1. Introduction

A thorough comprehension of the three-dimensional static structure of proteins is imperative for accurately understanding their functional roles. A fundamental exemplification of this is seen in photosynthesis, where solar radiation is converted into storable chemical energy [1,2,3,4,5].

The high-resolution structures of photosynthetic pigment-protein complexes have been predominantly derived through X-ray crystallography. For instance, the crystal structures of Photosystem I (PSI) have been acquired via electron crystallography at cryogenic temperatures [6,7]. More recently, XFEL crystallography provided crystal structures of PSI [8] and of Photosystem II (PSII) [9] even at room temperature. Subsequently, the structures of all intermediates of the Kok cycle representing the different steps of photosynthetic water splitting in PSII [10] were also reported from XFEL crystallography. Furthermore, cryo-EM experiments were used to determine a high-resolution structure of PSI [11], revealing deviations from (low temperature) crystal structures by an expansion of PSI in solution. Despite the invaluable structural information garnered from high-resolution methodologies in general, these techniques possess certain limitations rooted in the necessity for crystallization or the employment of low temperatures. This is directly inferred from the discrepancies noted between low-temperature and room temperature crystal structures (as demonstrated by XFEL crystallography, see above), as well as from the solution state (as illustrated by cryo-EM above). Consequently, it is typically unavoidable to ascertain the relevance of high-resolution structures for solubilized photosystems under physiological conditions [12,13,14,15]. The necessity of crystallization or the utilization of low temperatures in high-resolution structural biology also impinges on the feasibility to examine dynamical biological processes encompassing large-scale structural alterations or complex formation [11,16,17].

At this juncture, small-angle neutron and X-ray scattering (SANS and SAXS, respectively) may bridge this gap by furnishing structural information in solution and at physiological temperatures [18,19,20]. In turn, conformational dynamics [21,22], as well as vibrational properties of biomolecules [23,24] may be studied by neutron spectroscopy. SANS and SAXS have been previously utilized across diverse research domains such as the examination of food colloids [25], the advancement of mRNA vaccines [26], and in photosynthesis research (see [27,28] for reviews). The recent advancement of time-resolved SANS (TR-SANS) enables exploring dynamic processes within structural biology [29].

Within photosynthesis research, the primary utilization of SANS and SAXS has been in the studies concerning: (a) the oligomerization state of pigment-protein complexes; (b) protein-detergent complexes when solubilized membrane proteins are in buffer solution; and (c) large-scale structural alterations in proteins in response to external triggers. Pertaining to the first, information regarding the oligomerization state in solution for the cyanobacterial light-harvesting complexes phycocyanin, and for monomeric and trimeric PSI, has been supplied through SANS and SAXS. As to the second, many photosynthetic pigment protein complexes are transmembrane proteins and can only be extracted from the membrane by solubilization using amphipathic detergent molecules [30] to prevent unspecific aggregation [14,15,31]. This implies that a detergent belt encircles isolated membrane proteins in buffer solution, a feature not discernible in crystal structures due to its intrinsic structural heterogeneity. In this regard, the solution structures of detergent-protein complexes were determined for the major light-harvesting complex LHC II of green plants [15,32], the bacterial light-harvesting complex LH2 at physiological temperatures [33], of PS I [34,35,36], and PSII [37] using SANS and SAXS measurements. Lastly, the most distinctive potential for employing SANS and SAXS resides in probing large-scale structural alterations upon external triggers, as exemplified by the case of the orange carotenoid protein (OCP) [11,16,17]. These structural rearrangements are often elusive to high-resolution methods e.g., owing to their transient nature or the obstruction of large-scale motions by crystallization or cooling.

Despite the substantial corpus of literature concerning the exploration of solution structures in photosynthesis research via SANS and SAXS, the full potential of these methods remains to be harnessed. Predominantly, the limitations arise from the low resolution attainable and the ensuing challenge of correlating the structural data acquired from SANS and SAXS to high-resolution structures. Recent developments in modeling tools, along with a potential combination with molecular dynamics simulations, are poised to enhance the utility of SANS and SAXS, positioning them as complementary methodologies to X-ray crystallography and cryo-EM.

In the current review, developments in SAXS/SANS, particularly on photosynthetic protein complexes, will be described. Initially, a brief overview of the fundamental theory of SAXS and SANS is provided, complemented with illustrative model calculations. Subsequently, a review of chosen applications of SAXS and SANS to photosynthetic pigment protein complexes is presented. In conclusion, more recent advancements are discussed, anticipated to markedly amplify the impact of SAXS/SANS studies, comprising four subsections: (i) progress in modeling of SAXS/SANS data; (ii) the employment of selective deuteration in SANS with contrast variation; (iii) prospects for an amalgamation of SANS with molecular dynamics simulations; and (iv) direct integration of SANS with functional studies for the examination of structure-function relationships. We end with an overview about recent time-resolved SANS/SAXS experiments.

## 2. Basic SANS/SAXS Theory

We will now compile the most necessary theoretical expressions needed to analyze SAXS/SANS data. For a better understanding of the principle of both methods, we will also supply some illustrative model calculations at the end of this chapter. Briefly, SANS and SAXS are complementary experimental techniques that measure the form factor P(q) representing the size and shape of a biomolecule in an aqueous solution at physiological temperatures as a function of the scattering vector q [18,20]. The latter is related to the scattering angle 2θ and to the wavelength of the incident quasi-monochromatic radiation λ_0_ by the relation

(1) $$q=\frac{4\pi }{\lambda_{0}}\sin(\theta ),$$

Then, the intensity measured in a SANS experiment as a function of the scattering vector q is related to the form factor P(q) by the master equation [18,20,36]

(2) $$I\left(q\right)=n\Delta \rho^{2}V^{2}P\left(q\right)S\left(q\right),$$

where n is the particle number density, ∆ρ is the difference in scattering length density (SLD) between the particles and the solvent defining the contrast, and V is the volume of the particles. The effective structure factor S(q) is assumed to be equal to unity in the case of a diluted solution of monodisperse particles. P(q) describes the scattering from a single particle and depends on its size, shape, and internal structure and fulfills the condition that P(0) = 1. The mathematical expression for P(q), which is the space average for all possible particle orientations, can be written as:

(3) $$P\left(q\right)={\left|\frac{1}{V}\int \rho (r)e^{-iqr}dr\right|}^{2},$$

where ρ(r) is electron or nuclear density in the case of X-ray or neutron scattering, respectively. From the Formula (3), P(q) can be calculated for many particles with defined geometrical shapes of spheres, cylinders, ellipsoids etc. [38].

A model-independent analysis of small-angle scattering data applies the Guinier law [39], which is widely used to determine the basic particle parameters, such as radius of gyration (R_g_) and molecular weight (MW). The Guinier approximation is valid for diluted solutions of monodisperse particles and at small q values satisfying the condition qR_g_ < 1.3 [39], when the formula for P(q) can be rewritten as

(4) $$P\left(q\right)\sim {\left|\frac{1}{V}\int \rho (r)dr\right|}^{2}\left(1-q^{2}\frac{R_{g}^{2}}{3}+\ldots \right),$$

The integration of the right part of the formula corresponds to the forward scattering intensity I(0), and the other part in the bracket can be written in exponential form. Therefore, this approximation leads to a relatively simple relation between the measured intensity and the radius of gyration according to

(5) $$I\left(q\right)=I\left(0\right)\exp\left(-q^{2}\frac{R_{g}^{2}}{3}\right),$$

I(0) is the forward scattering, which is a shape-independent function of the total scattering power of the sample. Literally, I(0) is the sum of the scattering lengths of all atoms inside the particle. If the chemical composition of a particle is known, the evaluation of I(0) allows the molecular mass of the particle to be determined in both X-ray and neutron experiments.

If SAS data are normalized to the absolute values and the concentration of the protein is known, one can estimate the MW as [40]:

(6) $$MW=(\frac{N_{A}I\left(0\right)}{c})/∆\rho_{M}^{2},$$

N_A_ is the Avogadro number, c is the protein concentration, and Δρ_M_ is the scattering contrast per mass [40].

It is important to remember the limitations of the method. For instance, I(0) cannot be measured experimentally, so the extrapolation to the zero value of q must be used. In some cases obtaining an estimate of protein concentration is challenging, and the typical accuracy of the MW determination in practice is about 10–15% [40,41].

The scattered intensity can also be related to a function P(r) describing the distribution of pair distances of all atoms in a biomolecule. In the case of monodisperse macromolecular solutions, the scattering intensity is proportional to the scattering of a single particle averaged over all orientations is given by a Fourier transformation following

(7) $$I\left(q\right)=4\pi \int_{0}^{D_{max}}P\left(r\right)\frac{\sin(qr)}{qr}dr,$$

where P(r) is the pair distance distribution function, D_max_ corresponds to the maximum distance in the particle. The P(r) function and the particle maximum dimension D_max_ can be determined using the Inverse Fourier transform (IFT) method employing the software routine Gnom [42].

**Figure 1:**
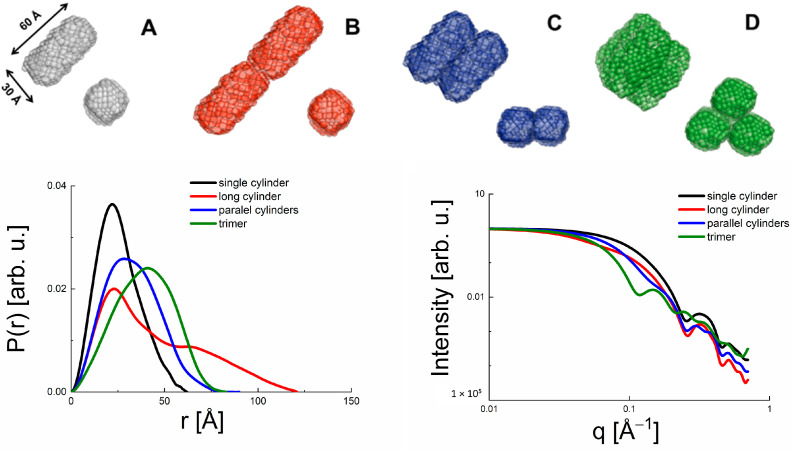
Top: Illustrative model systems consisting of approximate cylinders with a diameter of 30 Å and a length of 60 Å (from left to right): (**A**) monomer, (**B**) long dimer, (**C**) compact dimer and (**D**) trimer. Bottom, left: P(r) functions calculated from the model systems. Bottom, right: SAXS data calculated from the same model systems and normalized to I(0) = 1. The color scheme is kept throughout all figure parts.

The principle of deriving structural information from SAXS/SANS data is illustrated in Figure 1 by using four model protein systems based on an approximated monomeric cylinder with a diameter of 30 Å and a length of 60 Å. From left to right, a monomer, a long dimer, a compact dimer, and a trimer are constructed from the same basic unit. The resulting P(r) functions calculated from the model systems are shown on the left bottom, the SAXS data calculated from the same model systems on the right bottom of Figure 1. The P(r) function of the monomer has a peak at about 30 Å and extends to a maximum length D_max_ of a bit more than 60 Å. The latter values appear to be intuitive for the given monomer structure. It is now instructive to follow the P(r) functions of the other oligomers. The long dimer retains the main peak at 30 Å characterizing the two monomer structures within the dimer, but also exhibits a second distribution of higher radii above a length of 60 Å accounting for the extended dimer structure. The compact dimer, in contrast, has a P(r) function shifted to generally higher values, but not as high as the D_max_-value of the long dimer. This effect becomes even more pronounced for the even bigger compact trimer system. The resulting SAXS data can then be calculated according to Equation (4). Here, the curve of the (smallest) monomer structure is found at the highest q-values, since SAXS data are obtained in the Fourier space. Consequently, the higher-order oligomers (dimers, trimers) appear to be shifted to generally smaller q-values. It is interesting to note that there are subtle differences between long and compact dimer, though, which allow to distinguish their shapes in an experiment.

**Figure 2:**
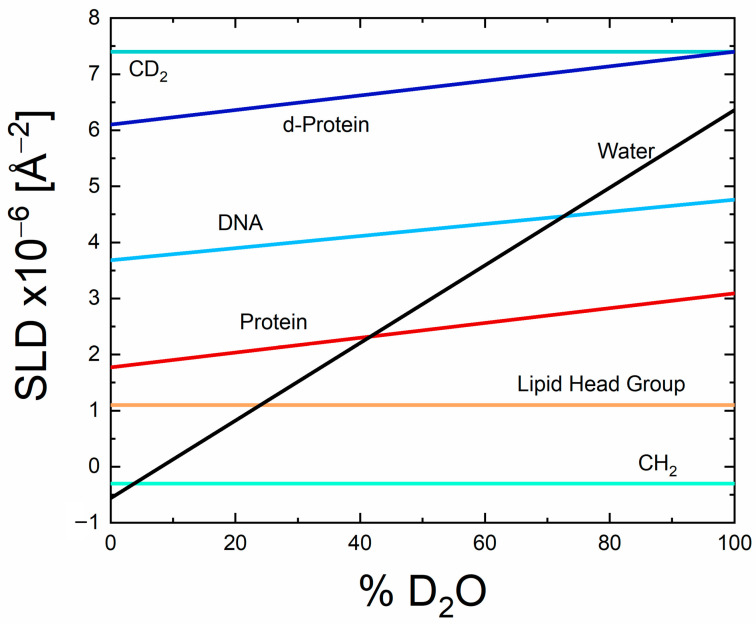
Illustration of contrast variation SANS experiments: variations of the SLD with increasing D_2_O content of the solvent for selected sample systems common in structural biology: proteins (red line), deuterated proteins (blue), DNA (light blue), lipids/detergents (light brown), CH_2_- and CD_2_-groups. The SLD of water (black line) increases drastically with increasing D_2_O content. Each crossing point of the black line with another SLD line indicates a specific D_2_O content (“match point”) where a specific constituent of a multi-component system cannot be distinguished from the solvent and is, thus, “matched out” in a SANS experiment.

While both SANS and SAXS techniques provide information about size and shape of biomolecules, their nature of interaction with a sample is different. Neutrons interact with the nuclei of a biomolecule, while X-rays get scattered by electrons. Due to the sensitivity of neutrons for different isotopes of the same atom, SANS has the special advantage of contrast variation by isotope exchange, e.g., the replacement of hydrogens by deuterium. Therefore, SANS is a useful tool to study multi-component protein complexes with selective deuterium labeling of individual components. Moreover, SANS is a powerful technique to probe systems containing components with different scattering length densities e.g., lipid (or detergent)-protein systems. The structural information of each individual component can be probed by varying the D_2_O ratio in the solvent referred to as contrast variation or scattering length density matching. As examples, the match points for lipids/detergents, proteins and nucleic acids are about 5–25%, 40–45% and 65–70% of D_2_O respectively, see Figure 2 [43].

However, SAXS does not permit isotope contrast variation since isotopes such as hydrogen and deuterium have the same X-ray scattering length; SAXS has considerable advantages in other respects. SAXS instruments are more widely available for practical use and their photon flux is several orders of magnitude higher than that of SANS instruments. Despite the risk of radiation damage occurring in SAXS experiments, the small amount of sample required and short measurement time in comparison to SANS experiments make this technique quite attractive to study biological systems. According to the statistic of the Small Angle Biological Data Bank (SASBDB), 93% of deposited SAS entries were measured on synchrotron radiation facilities and only 2% were obtained using neutrons [44].

## 3. Applications and Recent Developments of SANS/SAXS

SANS/SAXS has been routinely applied for studies of oligomerization states of proteins in aqueous solutions. As an example, the trimeric and hexameric forms of the light-harvesting complex phycocyanin (PC) from *T. elongatus* were distinguished using SAXS [45]. PC is involved in efficient light-harvesting and excitation energy transfer to reaction center complexes in cyanobacteria [46,47,48]. Aggregation can be easily verified by Guinier plots and manifests itself by a deviation from expected linearity in the low q-region indicating the presence of large particles in the sample solution (see, for example, the case of LHCII [31]).

Another prototypical application of SANS with contrast variation is the determination of solution structures of membrane proteins solubilized in a belt of detergent molecules [19]. As already mentioned in the introduction, SANS with contrast variation was used in photosynthesis research e.g., to characterize the solution structure of the major light-harvesting complex LHC II of green plants [15], the bacterial light-harvesting complex LH2 [33], trimeric PS I [34,35,36], and PSII [37]. The latter applications of SANS/SAXS were reviewed in detail by Golub et al. [36].

In the following, we discuss the recent progress in SANS and SAXS studies taking into account four aspects with a significant potential for further development of the methods. First, there are novel modeling tools linking SANS and SAXS data with high-resolution structures, but also enable the development of structures for flexible proteins or complexes undergoing structural changes upon external triggers. Also, selective deuteration of biomolecules bears a great potential to improve the spatial selectivity of the method through SANS with contrast variation. The same is true for combinations of SANS with molecular dynamics simulations. Finally, functional studies on photosynthetic proteins may greatly benefit from a direct combination with structural studies using SANS and SAXS.

## 4. Modeling

**Figure 3:**
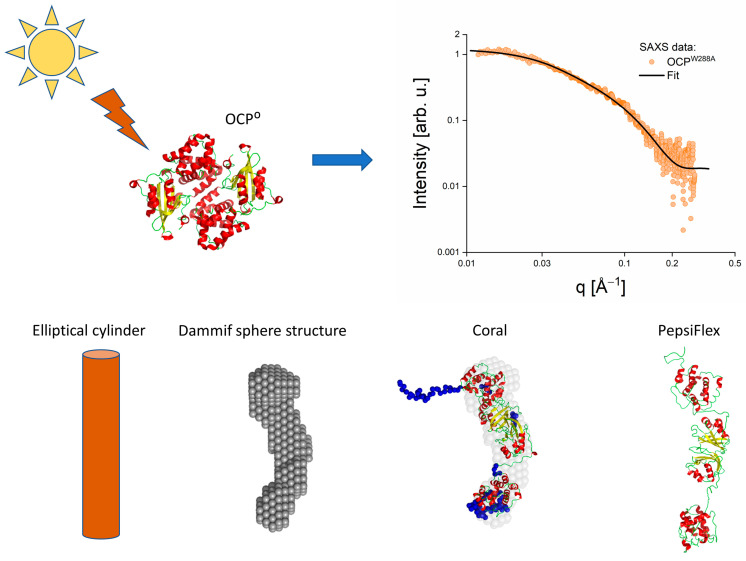
Top, left: The evolution of modeling development for SAXS/SANS data shown for the case of OCP (crystal structure of the ground state OCP^O^ pdb 3MG1 [49] shown in the upper left corner), Top, right: SAXS data of the mutant OCP^W288A^ assumed to be a structural analogue of the light-induced active state structure. Bottom: different models of the SAXS data shown in the upper right corner. SAXS data, DAMMIN and CORAL models taken from Golub et al. 2019 [16] with permission. The PepsiFlex structure was adapted from Golub et al., 2023 [50].

Although SAXS/SANS are low-resolution methods, recent advances in data modeling have significantly improved the opportunities of linking SAXS/SANS data to high-resolution structures available from crystallography or cryo-electron microscopy. Figure 3 illustrates different modeling approaches applied to the same SAXS curve of an OCP mutant conceived as an analogue of its active state OCP^R^. Generally, the spherical averaging of the SAXS/SANS scattering intensity caused by the random orientation of particles in solution leads to a considerable loss of information contained in the scattering data. Therefore, even if all of the particles in a solution under study are monodisperse, usually only some integral parameters can be evaluated from the scattering curve without a priori information. Below, we will list different methods of analyzing SAXS/SANS curves from isotropic monodisperse systems with special emphasis on recent developments.

The most straightforward analysis of small-angle data is the model-independent analysis of Guinier [39], which can estimate such parameters as the radius of gyration and I(0), which is related to the molecular mass of the studied system.

Another model-independent analysis approach applies the Inverse Fourier Transform (IFT) to calculate the pair distance distribution function P(r) from the small-angle scattering curve. The P(r) function represents the histogram of distances between atoms within the particle under study and can, therefore, guide which type of shape the particle has [42]. For monodisperse particles in solution, the scattering is proportional to the scattering of a single particle averaged over all orientations. One of the most widely used approaches to analyzing SAXS/SANS data is a “trial-and-error” modeling method. The experimental scattering intensity is compared with the scattering curves of some model bodies chosen based on a priori information on the particle.

The first attempts for the “trial-and-error” modeling were made with simple geometrical shapes such as spheres, cylinders, and ellipsoids, for which analytical equations to calculate scattering profiles are available. Such modeling gave only approximate information about protein dimensions as illustrated by the elliptical cylinder model in Figure 3.

Over the last decades, the development of tools for analyzing SAXS/SANS data has made significant progress. An important step forward was made by the development of the ab-initio modeling tool DAMMIF [51], which builds a reconstituted structure based on the P(r) function (illustrated by grey spheres in Figure 3). DAMMIF has options to apply symmetry or asymmetry limitations such as oblate or prolate types of structures. In combination with a developed algorithm to exclude non-compact sphere structures and the opportunity to calculate an average solution, DAMMIF analysis has become the most popular tool for the data analysis of SAXS/SANS data. However, certain limitations remain, such as the inability to account for inhomogeneous scattering length density of a particle. It may also fail in the case of flexible proteins with partly unfolded (non-compact) structures.

In contrast to ab initio modeling, the routines CRYSON or CRYSOL [52]. Can be used to calculate SAXS/SANS data based on high-resolution structures available from crystallography or cryo-electron microscopy. However, this approach delivers satisfactory results only in the case when the protein in solution is identical to the high-resolution structure.

The next step in the recent developments for the analysis of SAXS/SANS data from flexible proteins was the CORAL (COmplexes with RAndom Loops) routine [41]. This software performs SAXS-based rigid body modeling of defined components of protein complexes, while other components such as interdomain linkers may be defined as flexible. CORAL translates and rotates the atomic models of the rigid domains linked together by artificial flexible linkers. These rearrangements are not entirely random: the distances between the N- and C-terminal portions of the subsequent domains belonging to one chain are constrained. A simulated annealing protocol is employed to find the optimal positions and orientations of available high-resolution models of domains and the approximate conformations of the flexible portions of the polypeptide chain(s). A CORAL structure derived from SAXS data of the mutant OCP^W288A^ is shown in Figure 3 and compared to a DAMMIN structure. Blues spheres are indicating the flexible linker residues. In addition, CORAL can take into account symmetry as a constraint. Nevertheless, the addition of artificial linkers limits the use of CORAL structures. As a result, CORAL structures cannot be used as a direct input for MD simulations. Moreover, SANS data cannot be modeled by CORAL.

Finally, flexible refinement of protein structure by normal mode deformation can be achieved using software routines such as SREFLEX [53], MuliFOXS [54], and PEPSI (Polynomial Expansions of Protein Structures and Interactions) [55], which permit to analyze both SAXS and SANS data of flexible proteins based on their crystal structure. The Pepsi program applies the Nyquist-Shannon-Kotelnikov sampling theorem, where a multipole expansion order is adapted to the model’s size and the experimental data’s resolution. Applying the cubic spline interpolation speeds up the running time of PEPSI. The FlexFit mode of PEPSI performs SAXS/SANS-based flexible modeling of protein complexes. Similar to CORAL, rigid protein parts can be reoriented with respect to the initial position to fit the small-angle scattering data. However, PEPSI does not require the application of artificial linkers to mimic the flexible loops of proteins and uses the native sequence of residues of a given protein. Figure 3 illustrates the evolution of modeling for the case of SAXS data of the mutant OCP^W288A^, comparing the cylinder model, a DAMMIF sphere structure, a CORAL model, and a PEPSI model (data and models taken from Golub et al., 2019 [16]). The latter comparison underlines the remarkable progress made in modeling of SAXS/SANS data in the recent years. The availability of the latter modeling tools enables not only a verification of existing high-resolution structures in solution state, but especially also a modelling of solution structures obtained after external triggers as in the case of OCP:

## 5. (Specific) Deuteration

**Figure 4:**
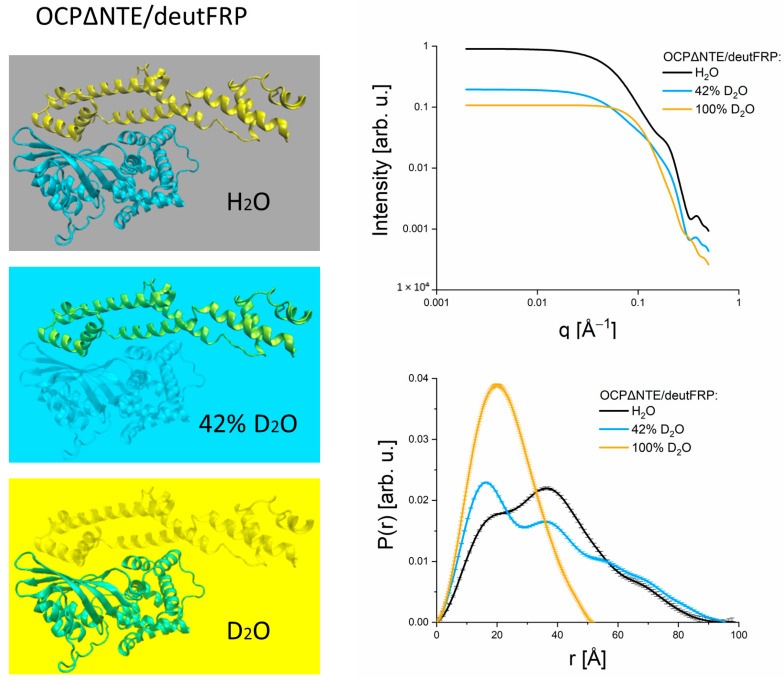
Left panels: Illustration of the principle of contrast matching for the structure of a complex of OCPΔNTE (blue) with deuterated FRP (yellow), structures are reproduced following Golub et al., 2023 [50]. The different matches achieved at contrasts of 0%, 42% and 100% D_2_O (from top to bottom) are indicated by different colors. Top, right: SANS data calculated for the complex of OCPΔNTE with deuterated FRP at the different contrasts. Bottom, right: P(r) functions calculated from the SANS data. The color scheme is kept throughout all figure parts.

An important issue for the full exploitation of the potential that SANS with contrast variation offers for structural studies of multicomponent systems in solution, is the need for deuteration of individual constituents of such multicomponent systems. Contemporary applications of selective deuteration for SANS studies have recently been summarized by Duff et al. [56]. This principle can be well applied to the case of complex formation out of different constituents. We illustrate this in Figure 4 using the example of the complex of the orange form of OCPΔNTE (blue) with deuterated FRP (yellow), structures are reproduced following Golub et al., 2023 [50]. The different matches achieved at contrasts of 0%, 42% and 100% D_2_O (from top to bottom) are indicated by different colors. At a contrast of 100% D_2_O, the deuterated FRP becomes completely matched so that the P(r) function obtained reflects that of OCP. The opposite is true for the contrast of 42% D_2_O, where only FRP is visible. Finally, at a contrast of 0%, the full complex can be studied. The latter approach allows to selectively assess the conformations of each constituent within the full complex and verify the possibility of conformational changes during complex formation. In the case of the OCP-FRP complex, the structures of two photocycle intermediates could be assigned by combining SANS with selective deuteration of complex partners [50] and optical spectroscopy [57].

**Figure 5:**
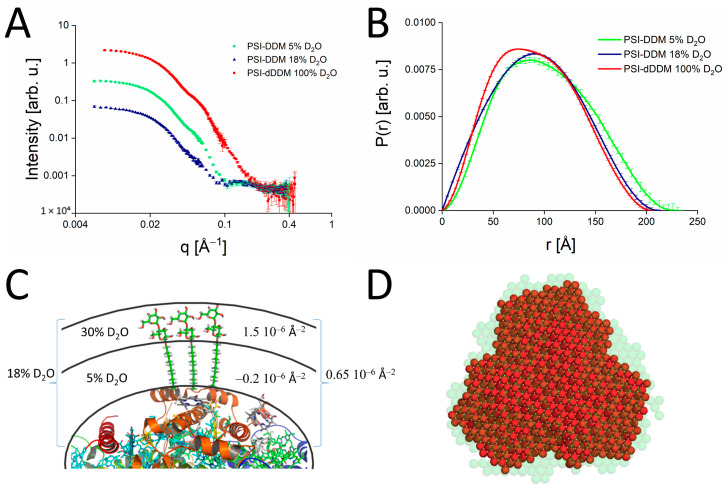
(**A**) SANS data of PSI obtained using specifically deuterated and protonated detergent are shown for the PSI-dDDM complex in 100% D_2_O (red points), the PSI-DDM complex in 18% D_2_O (navy), and the PSI-DDM complex in 5% D_2_O (green). The data of PSI at 5% D_2_O (green) are shown for comparison and taken from Kölsch et al. [11]. (**B**) Experimental P(R) functions corresponding to the SANS data shown in (**A**). (**C**) Scheme illustrating the principle of contrast matching of DDM bound at the surface of PSI, the SLD (right) and corresponding contrast match (left) are shown for the hydrophilic and hydrophobic groups as well as for the average of DDM. (**D**) Comparison of the structures obtained for the PSI-dDDM sample in 100% D_2_O (red spheres) and for the PSI-DDM sample in 5% D_2_O (light green spheres) reconstructed from the SANS data using the DAMMIN routine.

Another significant advancement in deuteration capabilities pertains to elucidating solution structures of membrane proteins solubilized within a surrounding belt of detergent molecules as first used by Midtgaard et al. [53]. This is discussed here using SANS data of the PSI-detergent complex shown in Figure 5A,B below. Until recently, the conventional approach for minimizing the contribution from detergent signals involved selecting a contrast match point based on the average SLD of the entire detergent molecule (see illustrating sketch in Figure 5C). In the case of DDM, this corresponded to approximately 18% D_2_O contrast. However, a closer inspection of Figure 5C reveals that the SLDs of the detergent head and tails are very different so that the average SLD does not yield a proper match for any of the detergent components. Therefore, proper matching of detergents requires a targeted specific deuteration of detergent head and tail groups. This specific deuteration results in the head and tail groups having identical SLDs, which can be accurately matched with a single contrast.

Specifically deuterated detergent has been successfully applied to study the solution structures of PSI-dDDM and PSII-dDDM complexes [31]. Figure 5A shows SANS data of PSI from *T. elongatus* acquired using regular DDM measured at two contrast points: 5% D_2_O (green dots) and 18% D_2_O (navy dots) corresponding to the match point of DDM tails and of DDM on average, respectively. The SANS curves are only roughly consistent with trimeric PSI [11,35,36,37], suggesting that conventional contrast matching of protonated detergents leads to seemingly larger complex sizes, as previously observed for PSI [11,35,36,37]. In contrast, the red dots in Figure 5A represent PSI isolated with the help of specifically deuterated dDDM measured at 100% D_2_O. The corresponding P(r) functions are given in Figure 5B. Comparing the experimental P(R) functions for all three SANS curves shown, one sees that the maximum particle dimension (D_max_) obtained from the PSI-dDDM SANS curve is 206.5 Å, while PSI-DDM curves at 5% and 18% D_2_O yield much larger D_max_-values of 230 Å and 215 Å, respectively.

The higher values of D_max_ in the case of PSI-DDM samples can be explained, taking into account the heterogeneous nature of the detergent, as illustrated in Figure 5C, since the contrast match points of detergent tails and head groups differ significantly. In the case of regular DDM, the head group’s SLD of about 1.5 10^−6^ Å^−2^ is positive, corresponding to a match point of 30% D_2_O. However, the SLD of the tail group of −0.2 10^−6^ Å^−2^ is negative, leading to a match point of 5% D_2_O. In the instance of the dDDM employed here, the deuteration levels for the hydrophobic and hydrophilic groups are 89% and 57%, respectively, resulting in an SLD of 6.36 10^−6^ Å^−2^, which can be almost perfectly matched out at 100% D_2_O [31,58].

Figure 5D compares the solution structures of the PSI-dDDM complex in 100% D_2_O (represented by red dots) and the PSI-DDM complex in 5% D_2_O (indicated by green spheres). These structures were derived from their P(r) functions using ab-initio reconstruction using the DAMMIN program. Notably, the length of a single detergent molecule is approximately 23 Å, roughly equivalent to the size of two spheres within the DAMMIN bead models shown in Figure 5D. The disparity between the two DAMMIN structures primarily amounts to 1–2 spheres. This discrepancy likely arises from an incomplete matching of the DDM belt that surrounds the protein in the case of DDM.

Furthermore, the distinction between the reconstructed structures implies that the solution structure of the PSI-DDM complex appears artificially enlarged, providing only a rough approximation of the native solution structure due to the incomplete matching of the DDM belt around the protein. In contrast, a proper detergent match is achieved only with the PSI-dDDM sample, resulting in a realistic representation of the solution structure. In summary, solution structures of membrane proteins involving detergent solubilization should routinely be measured by SANS using specifically deuterated detergents.

## 6. Combination with Molecular Dynamics Simulations

Despite the experimental advantages of SAXS/SANS, possible applications remain limited mainly due to the low resolution of the experimental data and due to a relative lack of theoretical tools to interpret the data. On the other hand, molecular dynamic (MD) simulations can predict the structure and dynamics of proteins at atomic resolution would certainly benefit from experimental benchmarking. The latter observations beg for a combination of the complementary techniques. Both SAXS/SANS and MD can be combined to validate the theoretical force field, the correctness of which is essential for MD, and overcome the low-resolution limit for SAXS/SANS structural models. For instance, the SAXS/SANS has already been successfully applied to verify the CHARMM36m force field for folded and intrinsically disordered proteins [59]. In that study, it was shown that the optimized CHARMM36m force field could predict the SAXS curve of the disordered arginine-serine peptide in water better than its previous iteration [59].

SAXS/SANS data give unique insight into the solution structure of proteins. MD simulations can complement the SAXS/SANS studies by modeling the flexibility between domains of proteins, which is often a key factor for its function. To note, proteins with large-scale flexibility, in general, are often not readily amenable to conventional structural analysis such as X-ray crystallography, nuclear magnetic resonance spectroscopy (NMR), or electron microscopy [60]. A combination of SAXS/SANS and MD has been applied to model flexible regions of the carotenoid-binding protein, which is the C-terminal homolog of OCP, and to optimize the conformational space of its C- and N-termini [61]. 

**Figure 6:**
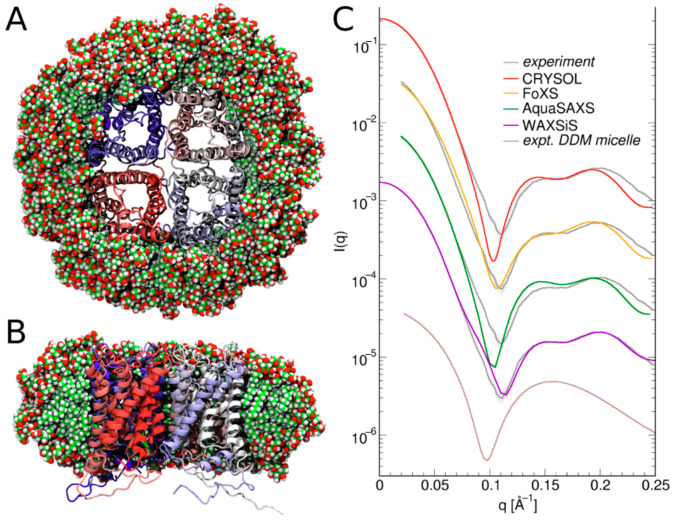
Illustration of the implicit and the explicit hydration layer approaches. An image of the 290-DDM protein-detergent complex and anticipated SAXS curves from four different predictors is presented. The protein-detergent complex is shown from the overhead perspective (**A**) and the lateral perspective (**B**), with the detergent omitted to display the structure of the lipid tail, as in reference [65]. DDM is depicted as spheres, while aquaporin-0 is illustrated in a cartoon style. (**C**) The projected SAXS intensities are color-coded, adjusted to match the experimental curve (in gray), and staggered vertically for better visualization. The software used is listed in the accompanying legend. A pure DDM micelle SAXS curve from reference [66] is also shown for comparison. The Figure is taken from reference [63] with permission.

Another aspect where the simultaneous application of SAXS/SANS and MD shows tremendous potential concerns the modeling of membrane proteins, solubilized by either detergent or nanodiscs in solution [62]. By employing MD simulation in the analysis of the SAXS data, Mirandela et al. were able to estimate the number of DDM detergent molecules in the detergent belt of the ammonium transporter AmtB from *Eschirichia coli* [62]. Notably, most software packages for calculating SAS profiles employ simplified, implicit models for the hydration layer. However, this approach introduces uncertainties into the calculations. For instance, as pointed out by Mirandela et al. [62], it is challenging to estimate the number of DDM detergent molecules within the detergent belt using the implicit hydration layer approximation framework. This limitation can be addressed through MD simulations, which enable the consideration of an explicit hydration layer. In this explicit model, the density of the hydration layer around biomolecules varies depending on the nature of the water-surface interactions. Such calculations can be performed using the WAXSis web platform, albeit at the expense of higher computational resources [63,64] (see Figure 6).

The above examples show that, despite of apparent intrinsic limitations [67], the combination of SAXS/SANS and MD has a great potential for a deeper understanding of solution structures and dynamics of protein complexes. It has to be added that solution structures obtained by PEPSI can be directly used as an input structure to derived force fields for MD simulations since it keeps the initial protein sequence.

## 7. Structure-Function Correlation

As outlined in the introduction, a major advantage of both SAXS and SANS is the possibility to perform structural studies in buffer solution, i.e., in nearly physiological conditions very similar to those employed in spectroscopic experiments to investigate functional properties of photosynthetic protein complexes. This leads to the opportunity to directly correlate structural information with protein function. This is especially important when the oligomerization state of the protein complex under investigation is not known or deviates from the corresponding crystal structure. Examples include the oligomerization state of PC [68] and phycobiliproteins (PBP) from cyanobacteria [45] and the light-harvesting complexes LH1 and LH2 from purple bacteria [33] and LHCII from green plants [32].

In the case of phycocyanin, Tsoraev et al. [68] showed using SAXS that the functional unit in their spectroscopic experiments was the PC trimer. Golub et al. [45] employed SANS to study PBP from *Acaryochloris marina* in different buffer solutions yielding different functional competence and made use of the fact that this light-harvesting complex is composed of known components: one allophycocyanin trimer (APC) and an unknown number of PC trimers. Complementary spectroscopic experiments had revealed that PBPs in buffer solution containing phosphate exhibit excitation energy transfer to the terminal emitter, while no such transfer is observed in phosphate-less buffer. 

**Figure 7:**
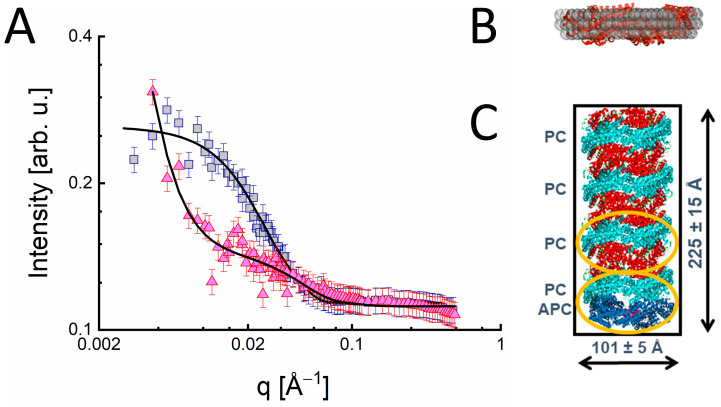
(**A**) SANS data of PBPs from *A. marina*, which are dissolved in buffers containing (PBP +P, blue squares) or lacking phosphate (PBP −P, red triangles), respectively. The full black lines are simultaneous fits of both data sets assuming a linear superposition of a cylinder (Equation (4)) and a power law (Equation (3)) accounting for aggregation in the case of PBP −P. (**B**) The crystal structure of PC from *T. elongatus* in trimeric form based on PDB code 4Z8K [71]. The grey spheres show the low-resolution structure of PC obtained using the program DAMMIF from the SAXS data shown in Figure 5. (**C**) schematic representation of PBPs from *A. marina* as a cylinder (black rectangle) based on the SANS data of the present study. The crystal structures of seven PC (red and turquoise) trimers (see [71], PDB code 4Z8K) and one APC (blue) trimer (see [72], pdb code 1B33) are superimposed on the cylinder structure, see text. The approximate sizes of one PC homodimer and one PC/APC heterodimer are indicated by orange ellipses. The images are taken from Golub et al., 2017 [45] with permission.

The SANS data shown in Figure 7A can be described well using a cylindrical shape with a length of about 225 Å and a diameter of approximately 100 Å. This finding is qualitatively consistent with earlier electron microscopy studies reporting a rod-like shape of the phycobiliproteins with a length of about 250 [69] or 300 Å [70]. In contrast, phycobiliproteins dissolved in buffer lacking phosphate revealed a splitting of the rods into cylindrical subunits with a height of 28 Å only, but also a pronounced sample aggregation. Complementary small SANS experiments suggest that the cylindrical subunits may represent either trimeric phycocyanin or trimeric allophycocyanin (see Figure 7B). This is in agreement with the assumption that a phycobiliprotein rod with a total height of about 225 Å can accommodate seven trimeric phycocyanin subunits and one allophycocyanin (see Figure 7C), each of which having a height of about 28 Å. The structural information obtained by SANS/SAXS can be used to interpret variations in the low-energy region of the 4.5 K absorption spectra of phycobiliproteins dissolved in buffer solutions containing and lacking phosphate, respectively.

**Figure 8:**
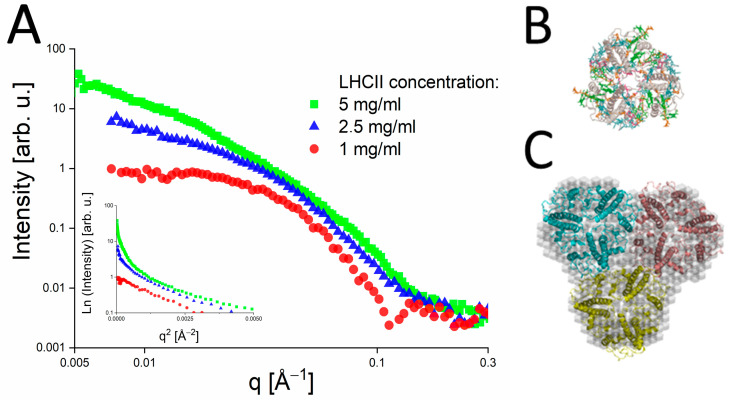
(**A**) SANS data of LHCII-β-DM complex obtained at different protein concentrations and at a contrast of 100% D_2_O. Green dots correspond to the measurement at a protein concentration of 5 mg/mL; blue dots—2.5 mg/mL and red dots—1 mg/mL. The inset shows the Guinier plot of the same SANS data. (**B**) The crystal structure of trimeric LHC II as obtained by Liu et al. [73]. (**C**) Structure reconstruction of LHCII–β-DM-sample based on SANS and SAXS data. The grey spherical structure is the reconstruction of the complex using DAMMIN. The images are taken from Golub et al., 2022 [32] with permission.

Tang et al. investigated the solution structures of bacterial light-harvesting complexes LH1 and LH2 from several different purple bacteria and based their analysis on the ring-shape of known crystal structures [33]. Golub et al. [32] found that different preparations of plant LHC II adopted different solution structures. The SANS data of the LHCII-βDM complex shown in Figure 8A reveal that only the lowest protein concentration of about 1 mg/mL is free of aggregation and exhibits a linear behavior in a Guinier plot. This suggests that the sample was prone to aggregation depending on the protein and detergent concentration The data of the LHCII-OG complex were consistent with the trimeric form of LHCII expected from the crystal structure (see Figure 8B), while the LHCII-βDM complex data yielded an unexpected nonameric form (see Figure 8C).

The observation of an oligomerization state deviating from that of the crystal structure implies the presence of unexpected pigment-pigment interactions between the different LHCII trimers discussed by Lambrev et al. [74], which may remain unnoticed without the SAXS/SANS experiments presented here. Lambrev et al. [74] concluded that trimer-trimer interactions may mimic the native organization of LHCII in the formation of energy-dissipative states for photoprotection. A potential candidate is neoxanthin protruding out of the LHCII crystal structure and being in close contact to Chl b606 and Chl a604. It is remarkable that Chl a604 was proposed to be a potential lowest energy state of LHCII [75,76] so that a photoprotective function via interaction with neoxanthin appears to be extremely physiologically relevant. The LHCII oligomer comprising three trimers shows that stable complexes mimicking trimer-trimer interactions are stable and can be used for spectroscopic studies.

The latter combined SANS/SAXS approach may thus provide a missing link to understand the photoprotective mechanism in LHCII.

## 8. Time-Resolved SANS/SAXS (TR-SANS/SAXS)

The continued development of time-resolved small-angle neutron scattering (TR-SANS) and of the corresponding instrumentation holds great promise for the future of structural biology in general. With the advancements in TR-SANS, we can expect to obtain more precise and detailed insights into dynamic biological processes involving larger scale conformational changes, enabling us better to understand the underlying mechanisms of many biological phenomena. As argued above, large-scale structural changes are usually not accessible to high-resolution methods since they are hindered by crystallization or cooling.

The latest advances in TR-SANS experiments permit the examination of the development of biological structures at a length scale ranging from nanometers to micrometers and over a timeframe that spans from seconds to days [29]. The duration of each time frame is dependent on various factors, such as the incoming neutron flux, collimation length, sample volume, sample concentration and contrast, incoherent background, and detector efficiency.

**Figure 9:**
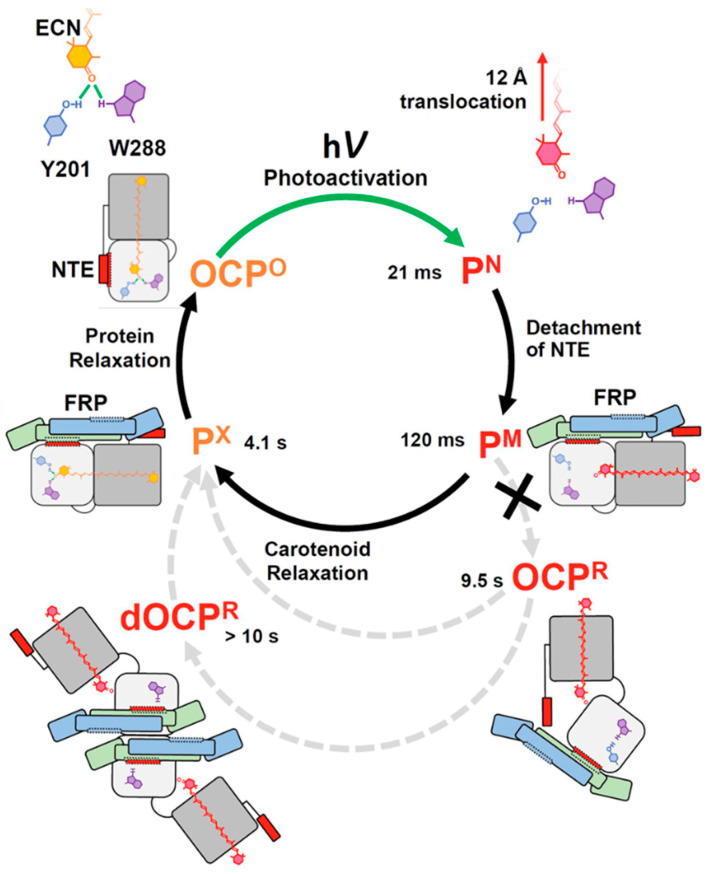
Scheme illustrating the photocycle of OCP when FRP is present, as described by Tsoraev et al. [57]. The red and orange symbols denote the intermediate states, the time values represent their relaxation times. OCP is represented by white and grey squares corresponding to the CTD and NTD domains, respectively. The chain-like molecule inside of the OCP domains is the carotenoid shown in the color of the specific intermediate. The Figure is reproduced from reference [57] with permission.

The supplementary sample environment must be adjusted to initiate the biochemical or biological process simultaneously with the commencement of neutron acquisition in the TR-SANS experiment. An example of such a biological process is the photocycle of the OCP, where the time scales of the intermediates are well-documented (see Figure 9) [57]. Therefore, this method goes beyond e.g., static SANS/SAXS experiments with in-situ illumination [16]. In TR-SANS, different triggering events, including fast mixing [77], temperature changes [78,79], and photo-spectroscopic methods [80], have been employed.

TR-SANS was demonstrated to be a valuable tool in studying the kinetics of protein aggregation and fibrillation. This technique is particularly well-suited for analyzing these processes due to the substantial scattering signal generated by the voluminous and extended structures involved and the considerable changes in aggregation numbers that occur. Notably, TR-SANS can detect observable changes in parameters such as I(0) and Rg during these processes, adding to its effectiveness in this field of study [29].

A second category of systems that TR-SANS routinely examines involves membrane structures and their dynamics, which are located at the intersection of biology and soft matter. Examples of these include lipid exchange processes in vesicles [81,82], the impact of phospholipidase on liposomes [83], the formation of liposomes in bile salts [84], lipid transfer between solid and free-standing membranes [85], and the dynamic interaction of peptides with membranes that mimic *Escherichia coli* [86].

TR-SANS can also investigate cellular ultra-structures. Numerous SANS studies on thylakoids have demonstrated changes in orientation and inter-membrane distances depending on external stimuli such as light illumination applied over a timeframe of seconds to minutes [87,88]. TR-SANS, combined with a specialized sample environment, has also been utilized effectively to investigate the effects of chemical and thermal treatment on cellulose and lignin biomasses [89,90,91,92].

Finally, TR-SANS can also be exploited to examine the conformational kinetics of individual bio-macromolecular structures and assemblies, although this approach is more challenging than the previous categories. The smaller bio-macromolecule size, the absence of periodic structural features, and lower concentrations of fibrils, membrane structures, and cellular ultra-structures make this approach more demanding. Still, TR-SANS has demonstrated excellent results in some studies, such as investigating the assembly process of the 800 kDa GroELGroES chaperonin from the archaeon *Thermoplasma acidophilum* [93] and the structural dynamics during the active unfolding and degradation process of the archaeal PAN-proteasome complex and GFP [78,79].

There is a large potential to apply TR-SAXS to photosynthetic and other photoactive proteins undergoing large-scale structural changes upon external (light) triggers. Examples include rhodopsin [94], PYP [95], the photocycle of OCP revealing structural changes between dark-adapted and active state, but also variations in oligomerization state and formation of oligomers [17].

To conclude, encouraging developments in biological TR-SANS experiments are underway. One of the areas of focus for improving TR-SANS experiments is the development of new instrumentation. The European Spallation Source in Lund is currently developing advanced instrumentation to reduce the amount of sample required. By minimizing the required sample amount, the new instrumentation will reduce the cost and time needed for TR-SANS experiments, making the technique more accessible to researchers. In addition, developing new instruments will broaden the range of accessible time scales, providing more precise and detailed information about dynamic biological processes. Another aspect of improving TR-SANS experiments is the enhancement of the sample environment. For TR-SANS to produce accurate and meaningful results, the ancillary sample environment must be appropriately adjusted to trigger the biochemical or biological process parallel to the start of neutron acquisition in the TR-SANS experiment. Developing a more specialized sample environment will allow for the more precise triggering of these processes, further improving the accuracy and sensitivity of TR-SANS measurements.

## Data Availability

The datasets generated during and/or analyzed in the present study are available from the corresponding author on reasonable request.

## References

[1] Nelson N., Yocum C.F. (2006). Structure and Function of Photosystems I and II. Annu. Rev. Plant Biol..

[2] Golbeck J.H. (2006). Photosystem I, the Light-Driven Plastocyanin: Ferredoxin Oxidoreductase.

[3] Müh F., Zouni A. (2011). Light-Induced Water Oxidation in Photosystem II. Front. Biosci..

[4] Lambreva M.D., Russo D., Polticelli F., Scognamiglio V., Antonacci A., Zobnina V., Campi G., Rea G. (2014). Structure/Function/Dynamics of Photosystem II Plastoquinone Binding Sites. Curr. Protein Pept. Sci..

[5] Najafpour M.M., Renger G., Holynska M., Moghaddam A.N., Aro E.M., Carpentier R., Nishihara H., Eaton-Rye J.J., Shen J.R., Allakhverdiev S.I. (2016). Manganese Compounds as Water-Oxidizing Catalysts: From the Natural Water-Oxidizing Complex to Nanosized Manganese Oxide Structures. Chem. Rev..

[6] Jordan P., Fromme P., Witt H.T., Klukas O., Saenger W., Krauss N. (2001). Three-Dimensional Structure of Cyanobacterial Photosystem I at 2.5 a Resolution. Nature.

[7] Mazor Y., Borovikova A., Caspy I., Nelson N. (2017). Structure of the Plant Photosystem I Supercomplex at 2.6 a Resolution. Nat. Plants.

[8] Gisriel C., Coe J., Letrun R., Yefanov O.M., Luna-Chavez C., Stander N.E., Lisova S., Mariani V., Kuhn M., Aplin S. (2019). Membrane Protein Megahertz Crystallography at the European Xfel. Nat. Commun..

[9] Young I.D., Ibrahim M., Chatterjee R., Gul S., Fuller F., Koroidov S., Brewster A.S., Tran R., Alonso-Mori R., Kroll T. (2016). Structure of Photosystem II and Substrate Binding at Room Temperature. Nature.

[10] Kern J., Chatterjee R., Young I.D., Fuller F.D., Lassalle L., Ibrahim M., Gul S., Fransson T., Brewster A.S., Alonso-Mori R. (2018). Structures of the Intermediates of Kok’s Photosynthetic Water Oxidation Clock. Nature.

[11] Kölsch A., Radon C., Golub M., Baumert A., Bürger J., Mielke T., Lisdat F., Feoktystov A., Pieper J., Zouni A. (2020). Current Limits of Structural Biology: The Transient Interaction between Cytochrome C6 and Photosystem I. Curr. Res. Struct. Biol..

[12] le Maire M., Champeil P., Möller J.V. (2000). Interaction of Membrane Proteins and Lipids with Solubilizing Detergents. Biochim. Biophys. Acta.

[13] Seddon A.M., Curnow P., Booth P.J. (2004). Membrane Proteins, Lipids and Detergents: Not Just a Soap Opera. Biochim. Biophys. Acta.

[14] Mo Y., Lee B.K., Ankner J.F., Becker J.M., Heller W.T. (2008). Detergent-Associated Solution Conformations of Helical and Beta-Barrel Membrane Proteins. J. Phys. Chem. B.

[15] Cardoso M.B., Smolensky D., Heller W.T., O’Neill H. (2009). Insight into the Structure of Light-Harvesting Complex II and Its Stabilization in Detergent Solution. J. Phys. Chem. B.

[16] Golub M., Moldenhauer M., Schmitt F.J., Feoktystov A., Mandar H., Maksimov E., Friedrich T., Pieper J. (2019). Solution Structure and Conformational Flexibility in the Active State of the Orange Carotenoid Protein. Part I: Small-Angle Scattering. J. Phys. Chem. B.

[17] Andreeva E.A., Nizinski S., Wilson A., Levantino M., De Zitter E., Munro R., Muzzopappa F., Thureau A., Zala N., Burdzinski G. (2022). Oligomerization Processes Limit Photoactivation and Recovery of the Orange Carotenoid Protein. Biophys. J..

[18] Jacques D.A., Trewhella J. (2010). Small-Angle Scattering for Structural Biology—Expanding the Frontier While Avoiding the Pitfalls. Protein Sci..

[19] Breyton C., Gabel F., Lethier M., Flayhan A., Durand G., Jault J.M., Juillan-Binard C., Imbert L., Moulin M., Ravaud S. (2013). Small-Angle Neutron Scattering for the Study of Solubilised Membrane Proteins. Eur. Phys. J. E.

[20] Kikhney A.G., Svergun D.I. (2015). A Practical Guide to Small Angle X-Ray Scattering (SAXS) of Flexible and Intrinsically Disordered Proteins. FEBS Lett..

[21] Kühn P., Pieper J., Kaminskaya O., Eckert H.J., Lechner R.E., Shuvalov V., Renger G. (2005). Reaction Pattern of Photosystem II: Oxidative Water Cleavage and Protein Flexibility. Photosynth. Res..

[22] Pieper J., Renger G. (2009). Protein Dynamics Investigated by Neutron Scattering. Photosynth. Res..

[23] Pieper J., Trapp M., Skomorokhov A., Natkaniec I., Peters J., Renger G. (2012). Temperature-Dependent Vibrational and Conformational Dynamics of Photosystem II Membrane Fragments from Spinach Investigated by Elastic and Inelastic Neutron Scattering. Biochim. Biophys. Acta.

[24] Golub M., Rusevich L., Irrgang K.D., Pieper J. (2018). Rigid Versus Flexible Protein Matrix: Light-Harvesting Complex II Exhibits a Temperature-Dependent Phonon Spectral Density. J. Phys. Chem. B.

[25] Gilbert E.P. (2019). Small-Angle X-Ray and Neutron Scattering in Food Colloids. Curr. Opin. Colloid Interface Sci..

[26] Arteta M.Y., Kjellman T., Bertesaghi S., Wallin S., Wu X., Kvist A.J., Dabkowska A., Szekely N., Radulescu A., Berendzen J. (2018). Successful Reprogramming of Cellular Protein Production through Mrna Delivered by Functionalized Lipid Nanoparticles. Proc. Natl. Acad. Sci. USA.

[27] Nagy G., Garab G., Pieper J., Allakhverdiev S., Rubin A.B., Shuvalov V.A. (2014). Neutron Scattering in Photosynthesis Research. Contemporary Problems of Photosynthesis.

[28] Tiede D.M., Thiyagarjan P., Amesz J., Hoff A.J. (1996). Characterization of Photosynthetic Supramolecular Assemblies Using Small Angle Neutron Scattering. Biophysical Techniques in Photosynthesis.

[29] Martel A., Gabel F. (2022). Time-Resolved Small-Angle Neutron Scattering (TR-SANS) for Structural Biology of Dynamic Systems: Principles, Recent Developments, and Practical Guidelines. Methods Enzym..

[30] Müh F., Zouni A. (2008). Micelle Formation in the Presence of Photosystem I. Biochim. Biophys. Acta.

[31] Golub M., Gätcke J., Subramanian S., Kölsch A., Darwish T., Howard J.K., Feoktystov A., Matsarskaia O., Martel A., Porcar L. (2022). “Invisible” Detergents Enable a Reliable Determination of Solution Structures of Native Photosystems by Small-Angle Neutron Scattering. J. Phys. Chem. B.

[32] Golub M., Lokstein H., Soloviov D., Kuklin A., Wieland D.C.F., Pieper J. (2022). Light-Harvesting Complex II Adopts Different Quaternary Structures in Solution as Observed Using Small-Angle Scattering. J. Phys. Chem. Lett..

[33] Tang K.H., Blankenship R.E. (2012). Neutron and Light Scattering Studies of Light-Harvesting Photosynthetic Antenna Complexes. Photosynth. Res..

[34] O’Neill H., Heller W.T., Helton K.E., Urban V.S., Greenbaum E. (2007). Small-Angle X-Ray Scattering Study of Photosystem I-Detergent Complexes: Implications for Membrane Protein Crystallization. J. Phys. Chem. B.

[35] Le R.K., Harris B.J., Iwuchukwu I.J., Bruce B.D., Cheng X., Qian S., Heller W.T., O’Neill H., Frymier P.D. (2014). Analysis of the Solution Structure of Thermosynechococcus Elongatus Photosystem I in N-Dodecyl-Beta-D-Maltoside Using Small-Angle Neutron Scattering and Molecular Dynamics Simulation. Arch. Biochem. Biophys..

[36] Golub M., Kölsch A., Feoktystov A., Zouni A., Pieper J. (2021). Insights into Solution Structures of Photosynthetic Protein Complexes from Small-Angle Scattering Methods. Crystals.

[37] Golub M., Hussein R., Ibrahim M., Hecht M., Wieland D.C.F., Martel A., Machado B., Zouni A., Pieper J. (2020). Solution Structure of the Detergent-Photosystem II Core Complex Investigated by Small Angle Scattering Techniques. J. Phys. Chem. B.

[38] Pedersen J.S. (1997). Analysis of Small-Angle Scattering Data from Colloids and Polymer Solutions: Modeling and Least-Squares Fitting. Adv. Colloid Interface Sci..

[39] Guinier A., Fournet G. (1955). Small-Angle Scattering of X-Rays.

[40] Mylonas E., Svergun D.I. (2007). Accuracy of Molecular Mass Determination of Proteins in Solution by Small-Angle X-Ray Scattering. J. Appl. Crystallogr..

[41] Petoukhov M.V., Franke D., Shkumatov A.V., Tria G., Kikhney A.G., Gajda M., Gorba C., Mertens H.D., Konarev P.V., Svergun D.I. (2012). New Developments in the Atsas Program Package for Small-Angle Scattering Data Analysis. J. Appl. Crystallogr..

[42] Svergun D.I. (1992). Determination of the Regularization Parameter in Indirect-Transform Methods Using Perceptual Criteria. J. Appl. Crystallogr..

[43] Jacrot B. (1976). The Study of Biological Structures by Neutrons Cattering from Solution. Rep. Prog. Phys..

[44] Kikhney A.G., Borges C.R., Molodenskiy D.S., Jeffries C.M., Svergun D.I. (2020). Sasbdb: Towards an Automatically Curated and Validated Repository for Biological Scattering Data. Protein Sci..

[45] Golub M., Combet S., Wieland D.C.F., Soloviov D., Kuklin A., Lokstein H., Schmitt F.J., Olliges R., Hecht M., Eckert H.J. (2017). Solution Structure and Excitation Energy Transfer in Phycobiliproteins of Acaryochloris Marina Investigated by Small Angle Scattering. Biochim. Biophys. Acta.

[46] Theiss C., Schmitt F.J., Pieper J., Nganou C., Grehn M., Vitali M., Olliges R., Eichler H.J., Eckert H.J. (2011). Excitation Energy Transfer in Intact Cells and in the Phycobiliprotein Antennae of the Chlorophyll D Containing Cyanobacterium Acaryochloris Marina. J. Plant Physiol..

[47] Gryliuk G., Rätsep M., Hildebrandt S., Irrgang K.D., Eckert H.J., Pieper J. (2014). Excitation Energy Transfer and Electron-Vibrational Coupling in Phycobiliproteins of the Cyanobacterium Acaryochloris Marina Investigated by Site-Selective Spectroscopy. Biochim. Biophys. Acta.

[48] Pieper J., Rätsep M., Golub M., Schmitt F.J., Artene P., Eckert H.J. (2017). Excitation Energy Transfer in Phycobiliproteins of the Cyanobacterium Acaryochloris Marina Investigated by Spectral Hole Burning. Photosynth. Res..

[49] Wilson A., Kinney J.N., Zwart P.H., Punginelli C., D’Haene S., Perreau F., Klein M.G., Kirilovsky D., Kerfeld C.A. (2010). Structural Determinants Underlying Photoprotection in the Photoactive Orange Carotenoid Protein of Cyanobacteria. J. Biol. Chem..

[50] Golub M., Moldenhauer M., Matsarskaia O., Martel A., Grudinin S., Soloviov D., Kuklin A., Maksimov E.G., Friedrich T., Pieper J. (2023). Stages of Ocp-Frp Interactions in the Regulation of Photoprotection in Cyanobacteria, Part 2: Small-Angle Neutron Scattering with Partial Deuteration. J. Phys. Chem. B.

[51] Franke D., Svergun D.I. (2009). Dammif, a Program for Rapid Ab-Initio Shape Determination in Small-Angle Scattering. J. Appl. Crystallogr..

[52] Svergun D.I., Barberato C., Koch M.H.J. (1995). Crysol -a Program to Evaluate X-Ray Solution Scattering of Biological Macromolecules Form Atomic Coordinates. J. Appl. Crystallogr..

[53] Panjkovich A., Svergun D.I. (2016). Deciphering Conformational Transitions of Proteins by Small Angle X-Ray Scattering and Normal Mode Analysis. Phys. Chem. Chem. Phys..

[54] Schneidman-Duhovny D., Hammel M., Tainer J.A., Sali A. (2016). Foxs, Foxsdock and Multifoxs: Single-State and Multi-State Structural Modeling of Proteins and Their Complexes Based on SAXS Profiles. Nucleic Acids Res..

[55] Grudinin S., Garkavenko M., Kazennov A. (2017). Pepsi-SAXS: An Adaptive Method for Rapid and Accurate Computation of Small-Angle X-Ray Scattering Profiles. Acta Crystallogr. Sect. D Struct. Biol..

[56] Duff A.P., Cagnes M., Darwish T.A., Krause-Heuer A.M., Moir M., Recsei C., Rekas A., Russell R.A., Wilde K.L., Yepuri N.R. (2022). Deuteration for Biological SANS: Case Studies, Success and Challenges in Chemistry and Biology. Methods Enzym..

[57] Tsoraev G.V., Bukhanko A., Budylin G.S., Shirshin E.A., Slonimskiy Y.B., Sluchanko N.N., Kloz M., Cherepanov D.A., Shakina Y.V., Ge B. (2023). Stages of Ocp-Frp Interactions in the Regulation of Photoprotection in Cyanobacteria, Part 1: Time-Resolved Spectroscopy. J. Phys. Chem. B.

[58] Midtgaard S.R., Darwish T.A., Pedersen M.C., Huda P., Larsen A.H., Jensen G.V., Kynde S.A.R., Skar-Gislinge N., Nielsen A.J.Z., Olesen C. (2018). Invisible Detergents for Structure Determination of Membrane Proteins by Small-Angle Neutron Scattering. FEBS J..

[59] Huang J., Rauscher S., Nawrocki G., Ran T., Feig M., de Groot B.L., Grubmuller H., MacKerell A.D. (2017). Charmm36: An Improved Force Field for Folded and Intrinsically Disordered Proteins. Biophys. J..

[60] Pelikan M., Hura G.L., Hammel M. (2009). Structure and Flexibility within Proteins as Identified through Small Angle X-Ray Scattering. Gen. Physiol. Biophys..

[61] Dominguez-Martin M.A., Hammel M., Gupta S., Lechno-Yossef S., Sutter M., Rosenberg D.J., Chen Y., Petzold C.J., Ralston C.Y., Polivka T. (2020). Structural Analysis of a New Carotenoid-Binding Protein: The C-Terminal Domain Homolog of the Ocp. Sci. Rep..

[62] Mirandela G.D., Tamburrino G., Ivanovic M.T., Strnad F.M., Byron O., Rasmussen T., Hoskisson P.A., Hub J.S., Zachariae U., Gabel F. (2018). Merging in-Solution X-Ray and Neutron Scattering Data Allows Fine Structural Analysis of Membrane-Protein Detergent Complexes. J. Phys. Chem. Lett..

[63] Chen P.C., Hub J.S. (2015). Structural Properties of Protein-Detergent Complexes from SAXS and Md Simulations. J. Phys. Chem. Lett..

[64] Knight C.J., Hub J.S. (2015). Waxsis: A Web Server for the Calculation of SAXS/WAXS Curves Based on Explicit-Solvent Molecular Dynamics. Nucleic Acids Res..

[65] Koutsioubas A., Berthaud A., Mangenot S., Perez J. (2013). Ab Initio and All-Atom Modeling of Detergent Organization around Aquaporin-0 Based on SAXS Data. J. Phys. Chem. B.

[66] Lipfert J., Columbus L., Chu V.B., Lesley S.A., Doniach S. (2007). Size and Shape of Detergent Micelles Determined by Small-Angle X-Ray Scattering. J. Phys. Chem. B.

[67] Zec N., Mangiapia G., Hendry A.C., Barker R., Koutsioubas A., Frielinghaus H., Campana M., Ortega-Roldan J.L., Busch S., Moulin J.F. (2021). Mutually Beneficial Combination of Molecular Dynamics Computer Simulations and Scattering Experiments. Membranes.

[68] Tsoraev G.V., Protasova E.A., Klimanova E.A., Ryzhykau Y.L., Kuklin A.I., Semenov Y.S., Ge B., Li W., Qin S., Friedrich T. (2022). Anti-Stokes Fluorescence Excitation Reveals Conformational Mobility of the C-Phycocyanin Chromophores. Struct. Dyn..

[69] Chen M., Floetenmeyer M., Bibby T.S. (2009). Supramolecular Organization of Phycobiliproteins in the Chlorophyll D-Containing Cyanobacterium Acaryochloris Marina. FEBS Lett..

[70] Marquart A., Flockerzi V. (1997). Alpha1-Beta Interaction in Voltage-Gated Cardiac L-Type Calcium Channels. FEBS Lett..

[71] Conrad C.E., Basu S., James D., Wang D., Schaffer A., Roy-Chowdhury S., Zatsepin N.A., Aquila A., Coe J., Gati C. (2015). A Novel Inert Crystal Delivery Medium for Serial Femtosecond Crystallography. IUCrJ.

[72] Reuter W., Wiegand G., Huber R., Than M.E. (1999). Structural Analysis at 2.2 a of Orthorhombic Crystals Presents the Asymmetry of the Allophycocyanin-Linker Complex, Ap.Lc7.8, from Phycobilisomes of Mastigocladus Laminosus. Proc. Natl. Acad. Sci. USA.

[73] Liu Z., Yan H., Wang K., Kuang T., Zhang J., Gui L., An X., Chang W. (2004). Crystal Structure of Spinach Major Light-Harvesting Complex at 2.72 a Resolution. Nature.

[74] Lambrev P.H., Varkonyi Z., Krumova S., Kovacs L., Miloslavina Y., Holzwarth A.R., Garab G. (2007). Importance of Trimer-Trimer Interactions for the Native State of the Plant Light-Harvesting Complex II. Biochim. Biophys. Acta.

[75] Pieper J., Rätsep M., Irrgang K.D., Freiberg A. (2009). Chromophore-Chromophore and Chromophore-Protein Interactions in Monomeric Light-Harvesting Complex II of Green Plants Studied by Spectral Hole Burning and Fluorescence Line Narrowing. J. Phys. Chem. B.

[76] Pieper J., Irrgang K.D. (2020). Nature of Low-Energy Exciton Levels in Light-Harvesting Complex II of Green Plants as Revealed by Satellite Hole Structure. Photosynth. Res..

[77] Grillo I. (2009). Applications of Stopped-Flow in SAXS and SANS. Curr. Opin. Colloid Interface Sci..

[78] Ibrahim Z., Martel A., Moulin M., Kim H.S., Hartlein M., Franzetti B., Gabel F. (2017). Time-Resolved Neutron Scattering Provides New Insight into Protein Substrate Processing by a Aaa+ Unfoldase. Sci. Rep..

[79] Mahieu E., Coves J., Kruger G., Martel A., Moulin M., Carl N., Hartlein M., Carlomagno T., Franzetti B., Gabel F. (2020). Observing Protein Degradation by the Pan-20s Proteasome by Time-Resolved Neutron Scattering. Biophys. J..

[80] Chaudhari A.S., Semanat E.C., Martel A., Schneider B., Fuertes G. (2021). Light-Induced Oligomerization of the Transcription Factor El222. Acta Crystallogr. A-Found. Adv..

[81] Nakano M., Fukuda M., Kudo T., Endo H., Handa T. (2007). Determination of Interbilayer and Transbilayer Lipid Transfers by Time-Resolved Small-Angle Neutron Scattering. Phys. Rev. Lett..

[82] Nakano M., Fukuda M., Kudo T., Matsuzaki N., Azuma T., Sekine K., Endo H., Handa T. (2009). Flip-Flop of Phospholipids in Vesicles: Kinetic Analysis with Time-Resolved Small-Angle Neutron Scattering. J. Phys. Chem. B.

[83] Ferguson E.L., De Luca E., Heenan R.K., King S.M., Griffiths P.C. (2010). Time-Resolved Small-Angle Neutron Scattering as a Tool for Studying Controlled Release from Liposomes Using Polymer-Enzyme Conjugates. Macromol. Rapid Commun..

[84] Nawroth T., Buch P., Buch K., Langguth P., Schweins R. (2011). Liposome Formation from Bile Salt-Lipid Micelles in the Digestion and Drug Delivery Model Fassif(Mod) Estimated by Combined Time-Resolved Neutron and Dynamic Light Scattering. Mol. Pharm..

[85] Wah B., Breidigan J.M., Adams J., Horbal P., Garg S., Porcar L., Perez-Salas U. (2017). Reconciling Differences between Lipid Transfer in Free-Standing and Solid Supported Membranes: A Time-Resolved Small-Angle Neutron Scattering Study. Langmuir.

[86] Nielsen J.E., Prevost S.F., Jenssen H., Lund R. (2021). Impact of Antimicrobial Peptides on E. Coli-Mimicking Lipid Model Membranes: Correlating Structural and Dynamic Effects Using Scattering Methods. Faraday Discuss..

[87] Nagy G., Posselt D., Kovacs L., Holm J.K., Szabo M., Ughy B., Rosta L., Peters J., Timmins P., Garab G. (2011). Reversible Membrane Reorganizations During Photosynthesis in Vivo: Revealed by Small-Angle Neutron Scattering. Biochem. J..

[88] Nagy G., Kovacs L., Unnep R., Zsiros O., Almasy L., Rosta L., Timmins P., Peters J., Posselt D., Garab G. (2013). Kinetics of Structural Reorganizations in Multilamellar Photosynthetic Membranes Monitored by Small-Angle Neutron Scattering. Eur. Phys. J. E Soft Matter.

[89] Nishiyama Y., Langan P., O’Neill H., Pingali S.V., Harton S. (2014). Structural Coarsening of Aspen Wood by Hydrothermal Pretreatment Monitored by Small- and Wide-Angle Scattering of X-Rays and Neutrons on Oriented Specimens. Cellulose.

[90] Pingali S.V., O’Neill H.M., Nishiyama Y., He L.L., Melnichenko Y.B., Urban V., Petridis L., Davison B., Langan P. (2014). Morphological Changes in the Cellulose and Lignin Components of Biomass Occur at Different Stages During Steam Pretreatment. Cellulose.

[91] Pingali S.V., Smith M.D., Liu S.H., Rawal T.B., Pu Y.Q., Shah R., Evans B.R., Urban V.S., Davison B.H., Cai C.M. (2020). Deconstruction of Biomass Enabled by Local Demixing of Cosolvents at Cellulose and Lignin Surfaces. Proc. Natl. Acad. Sci. USA.

[92] Yang Z., Foston M.B., O’Neill H., Urban V.S., Ragauskas A., Evans B.R., Davison B.H., Pingali S.V. (2022). Structural Reorganization of Noncellulosic Polymers Observed in Situ During Dilute Acid Pretreatment by Small-Angle Neutron Scattering. ACS Sustain. Chem. Eng..

[93] Gutsche I., Holzinger J., Rauh N., Baumeister W., May R.P. (2001). Atp-Induced Structural Change of the Thermosome Is Temperature-Dependent. J. Struct. Biol..

[94] Perera S.M.D.C., Chawla U., Shrestha U.R., Bhowmik D., Struts A.V., Qian S., Chu X.Q., Brown M.F. (2018). Small-Angle Neutron Scattering Reveals Energy Landscape for Rhodopsin Photoactivation. J. Phys. Chem. Lett..

[95] Kim J.G., Kim T.W., Kim J., Ihee H. (2015). Protein Structural Dynamics Revealed by Time-Resolved X-Ray Solution Scattering. Acc. Chem. Res..

